# Telephone consultations for general practice: a systematic review

**DOI:** 10.1186/s13643-017-0529-0

**Published:** 2017-07-03

**Authors:** Martin J Downes, Merehau C Mervin, Joshua M Byrnes, Paul A Scuffham

**Affiliations:** 1 Centre for Applied Health Economics, School of Medicine, Griffith University, Nathan Campus - N78 1.11, 170 Kessels Rd, Nathan, Queensland 4111 Australia; 2 Menzies Health Institute Queensland, Griffith University, Nathan Campus - N78 1.11, 170 Kessels Rd, Nathan, Queensland 4111 Australia

**Keywords:** Telemedicine, Telehealth, General practice, Teleconsult

## Abstract

**Background:**

The use of information technology, including internet- and telephone-based resources, is becoming an alternative and supporting method of providing many forms of services in a healthcare and health management setting. Telephone consultations provide a promising alternative and supporting service for face-to-face general practice care. The aim of this review is to utilize a systematic review to collate evidence on the use of telephone consultation as an alternative to face-to-face general practice visits.

**Methods:**

A systematic search of MEDLINE, CINAHL, The Cochrane Library, and the International Clinical Trials Registry Platform was performed using the search terms for the intervention (telephone consultation) and the comparator (general practice). Systematic reviews and randomized control trials that examined telephone consultation compared to normal face-to-face consultation in general practice were included in this review. Papers were reviewed, assessed for quality (Cochrane Collaboration’s ‘Risk of bias’ tool) and data extracted and analysed.

**Results:**

Two systematic reviews and one RCT were identified and included in the analysis.

The RCT (*N* = 388) was of patients requesting same-day appointments from two general practices and patients were randomized to a same-day face-to-face appointment or a telephone call back consultation. There was a reduction in the time spent on consultations in the telephone group (1.5 min (0.6 to 2.4)) and patients in the telephone arm had 0.2 (0 to 0.3) more follow-up consultations than the face-to-face group.

One systematic review focused on telephone consultation and triage on healthcare use, and included one RCT and one other observational study that examined telephone consultations. The other systematic review focused on patient access and included one RCT and four observational studies that examined telephone consultations. Both systematic reviews provided narrative interpretations of the evidence and concluded that telephone consultations provided an appropriate alternative to telephone consultations and reduced practice work load.

**Conclusion:**

There is a lack of high level evidence for telephone consultations in a GP setting; however, current evidence suggests that telephone consultations as an alternative to face-to-face general practice consultations offers an appropriate option in certain settings.

**Systematic review registration:**

PROSPERO CRD42015025225

**Electronic supplementary material:**

The online version of this article (doi:10.1186/s13643-017-0529-0) contains supplementary material, which is available to authorized users.

## Background

Telephone consultation provides a promising alternative to face-to-face general practice (GP) care [1]. This seems particularly important in rural and remote areas where a sparse population means it is difficult to provide primary care in these regions without travelling long distances [2, 3]. However, while the published evidence has demonstrated that telemedicine is likely to be effective, there are inconsistencies in the available evidence [1].

Varying types of telemedicine and telephone consultations are available for GP consultations, specialist consultations and disease management in a number of countries. GP telephone consultations are currently being used in countries like the United Kingdom (UK), the Unites States of America (US), Denmark and Switzerland as an alternative to a face-to-face GP consultation and it has been suggested to provide timely care that is easily accessible [4–6]. Telephone consultations by general practices in the UK for ongoing patient care can be provided through the National Health Service (NHS) [5]. In Australia, telemedicine is currently available through a number of Medicare Benefits Schedule items for specialist services and disease management including videoconferencing by a specialist, consultant physician, telepsychiatry, consultant occupational physician, pain medicine physician, palliative medicine physician or neurosurgeon [7]. Telemedicine is also currently available worldwide for other services such as teleradiology, behaviour management support (smoking cessation), or remote monitoring for cardiovascular disease [1].

Telephone contacts have been considered similar to face-to-face contacts when used for health promotion, triage and providing long-term management for chronic diseases [8]. While there is some evidence available for telemedicine for management and monitoring in specific diseases there is a dearth of evidence for telephone consultation as an alternative for face-to-face general practice visits. A systematic review (of systematic reviews) in 2010 failed to identify any publications for telephone consultation as a replacement to general practice visits [1].

The aim of this study was to undertake a systematic review of the evidence on the use of telephone consultation as an alternative to general practice visits.

## Methods

The participants, interventions, comparators, and outcomes (PICO) for this systematic review were:

- Participants: people looking to access general practice services
- Intervention: telemedicine
- Comparator: normal care (face-to-face consultation),
- Outcomes: quality adjusted life years, hospitalization, emergency department use, mortality, time to treatment and other relevant service outcomes

A preliminary scoping search was conducted to identify terminology for the search terms and the type of studies that are likely to be available. This protocol has been reported according to the Preferred Reporting Items for Systematic Reviews and Meta-Analyses Protocols (PRISMA-P) guidelines [9] (Additional file 1) and is registered with PROSPERO (CRD42015025225). A detailed description of the analysis can be found at Downes et al. [10].

**Fig. 1:**
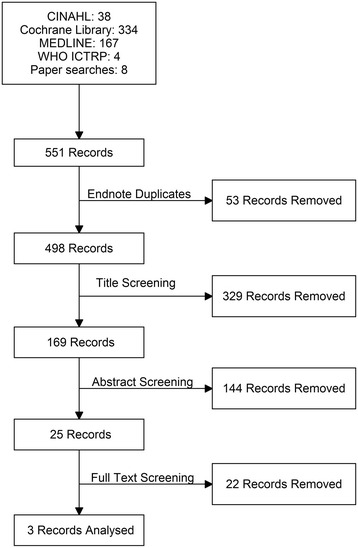
Flow diagram showing the total number of records identified and the number of records filtered at each stage of the selection process from the literature search of a systematic review on GP telephone consultation in 2015.

In summary MEDLINE, CINAHL, The Cochrane Library, International Clinical Trials Registry Platform and citation lists of included studies and relevant reviews were searched for relevant systematic reviews and randomized control trials on the 9 September 2015. Keywords and subject headings relating to telephone consultations in general practice were used for the searches (Additional file 2). For example CINAHL Plus was searched using: (Telemedicine OR Teleconsult OR “Tele* Consult*” or “*phone* Consult*” OR Telephone Consultation* OR Telehealth OR ehealth OR tele-health OR telemedicine) AND (General Practice OR Family Practi* OR primary health care OR family physician) AND (systematic review OR meta-analysis OR Randomized Controlled Trial OR RCT*). Then study selection was carried out by three of the authors, CM and JB selected studies independently; where difference in selection occurred between CM and JB, MD assessed those documents and selection was finalized by consensus. A PRISMA study flow chart demonstrates the inclusion exclusion process (Fig. 1). The following inclusion/exclusion criteria were used:

Inclusion criteria:

- The studies examined telephone consultations as an alternative to direct access to general practice
  - ○ The telephone consultation was patient initiated
  - ○ The telephone consultation was carried out by a general practitioner
- The studies followed up participants for health-related outcomes, and/or health care utilization
- The studies analysed primary data
- The studies were systematic reviews or randomized control trials (or in the case that none of these exists: The studies were cohort studies, case control studies and/or cross-sectional studies).

Exclusion criteria:

- The studies only examined telemedicine in specific disease populations
- The studies examined only telemonitoring or the use of telemedicine for the management of disease
- The studies examined only telemedicine used as follow-up that was initiated by the health practitioner
- The studies did not examine general practitioner-led telemedicine (i.e. nurse-led or specialist-led telemedicine)
- The studies’ outcomes were only patient satisfaction
- The publications were narrative reviews

### Data extraction

A standardized data extraction form was created for the data extraction process. Data extracted from the studies included information on the study characteristics, population baseline characteristics, the intervention, the comparator and outcomes. Critical appraisal of studies that were included was conducted using the Cochrane Collaboration’s ‘Risk of bias’ tool (Version 5.1.0.) [11].

### Analysis

The strength of the evidence was assessed using the Grading of Recommendations Assessment, Development and Evaluation (GRADE) guidelines to aid in the interpretation of the existing evidence and presenting recommendations for practice and future research [12].

## Results

### Search results

The combined searches of the bibliographic databases identified 551 records. A total of 53 duplicate records were found in the combined dataset, leaving 479 publications for consideration. After title, abstract and full text screening, three relevant papers were identified and included in the analysis (Fig. 1). During full text screening, two papers required assessment from the third reviewer and were excluded from the analysis.

### Included studies

Of the three studies identified for inclusion, one study was a randomized control trial (RCT) and two studies were systematic reviews that included the identified RCT in addition to other observational studies [13–15].

### Outcomes

McKinstry et al. [15] conducted a randomized control trial of 388 patients that requested same-day appointments from two general practices in an Edinburgh (UK) suburb. Patients were randomized to a same-day face-to-face appointment or a telephone call back for an over the telephone consultation. There was no prior triage; when a patient requested an appointment, they were either given a telephone or a face-to-face appointment for later that day. The key outcome for the trial was resource utilization. McKinstry et al. [15] found that there was a reduction in the time spent on consultation in the telephone group of 1.5 min (0.6 to 2.4). Secondary outcomes found that patients in the telephone consult group had 0.2 (0 to 0.3) more follow-up consultations than in the face-to-face group and were less likely to have blood pressure measured (Table 1). There were no other significant differences between the groups (Additional file 2). The risk of bias for McKinstry et al. is presented in Additional file 2.

**Table 1:** Evidence profile for a systematic review on telephone consultation in general practice

Quality assessment							Summary of findings					Importance
							No of patients		Effect		Quality	
No of studies	Design	Limitations	Inconsistency	Indirectness	Imprecision	Other considerations	Telephone	Face-to-face	Difference (95% CI)	Absolute		
Doctor time/min (SD)												
1^a^	Randomized trial	No serious limitations	NA	No serious indirectness	No serious imprecision	Publication bias a possibility	181	187	MD −1.5 (−2.4 to −0.6)	1.5 min less per patient (from 36 s less to 2.4 min less)	HIGH	CRITICAL
Subsequent GP contact (mean (SD), *N*)												
1^a^	Randomized trial	No serious limitations	NA	No serious indirectness	Some imprecision	Publication bias a possibility	182	188	MD 0.2 (0.0 to 0.3)	2 more visits per 10 people (from 3 more to no more)	HIGH	CRITICAL
BP measured (*n/N*)												
1^a^	Randomized trial	No serious limitations	NA	No serious indirectness	Some imprecision	Publication bias a possibility	12/181	25/188	RR 0.5 (0.26, 0.96)	7 less BPs measured per 100 (from 13 less to 6 less)	HIGH	IMPORTANT
Subsequent A&E contact (mean (SD), N)												
1^a^	Randomized trial	No serious limitations	NA	No serious indirectness	Some imprecision	Publication bias a possibility	182	188	MD 0.0^b^ (−0.1 to 0.0)	No more visits per 10 people	HIGH	CRITICAL

*MD* Mean difference, *RR* relative risk

^a^McKinstry et al. (2002)

^b^Not statistically significant

Bunn et al. was a systematic review that included many levels of scientific evidence with a specific focus on utilization. The systematic review included McKinstry et al. [15]. Bunn et al. identified one other observational study [16], that estimated a 39% reduction in the number of patients requiring face-to-face consultation, this was based on the number of patients that received a face-to-face consultation after the telephone consultation, and was not based on comparative estimates.

Chapman et al. [14] was a systematic review that also included many levels of scientific evidence; however, the focus of this review was on patient access. Both McKinstry et al. [15] and Jiwa et al. [16] were also included in this review along with three other observational studies [8, 17, 18]. Chapman et al. [14] concluded that both patients and health care providers considered the telephone as an appropriate means of communication and an appropriate alternative to a face-to-face appointment or home visits. Chapman et al. [14] also suggested that telephone consultations may lead to a decrease in demand for face-to-face consultations. Patient satisfaction with telephone consultations is dependent on ease of access to the GP and hence the patient-to-telephone line ratio of the practice is important.

## Discussion

This systematic review aimed to explore the efficacy of telephone consultations as an alternative to general practice face-to-face consultations. Only one randomized control trial and two systematic reviews were identified. The two systematic reviews identified the included trial along with other observational studies. Overall the included studies demonstrated that telephone consultations provide an appropriate alternative to face-to-face consultations. Although telephone consultations led to an increase in the number of repeated visits, there was still a reduction in time spent with patients overall.

The systematic reviews presented additional evidence from observational studies that examined telephone GP consultations. However, the observational studies were limited by small sample size and a lack of comparison to usual care. Therefore, it is difficult to interpret the outcomes from these studies. In general the included observational studies tended to agree with the higher level evidence showing a similar degree of patient satisfaction with GP telephone consults [18] and strengthening the argument that the telephone consult was appropriate in certain situations [15]. Car and Sheikh [8] indicate that these situations could encompass a broad spectrum of problems and listed, management of urinary tract infections in women, monitoring for depression; management of diabetes, counselling for smoking cessation, among others as having good evidence.

The included randomized control trial recruited patients directly from the reception of two general practices and no triage was carried out, this is normal for general practices that use telephone consultations in the UK. However, some GP telephone systems operate through a triage system, where patients are first assessed by a nurse and then assigned to a face-to-face consultation or a telephone consultation [13, 14, 19, 20]. The triage system may provide a further benefit in reducing work load in a general practice setting, as it will streamline the process and reduce the number of patients receiving a telephone consultation where a face-to-face consultation would have been more appropriate. However, this may not be the case when GPs conduct the triage as Campbell et al. [20] concluded that the number of GP contacts per person is increased when GP triage was compared to usual care.

Due to the diversity of a GP consultation and the exhaustive lists of common presentations [21], it is difficult to identify efficacy outcomes to compare modes of consultation delivery. Studies of the GP consultation often utilize patient satisfaction as their preferred outcome of interest [13, 14]. Diagnostic agreement has been used as a measure of the benefit of different modes of consultation delivery in a GP setting [22]; however, utilizing this outcome requires a crossover trial design which includes inherited biases. Dixon and Stahl [22] noted that the level of agreement was similar between the face-to-face GP and virtual visit (84%) compared with between face-to-face with one doctor and face-to-face with a different doctor (80%). Diagnostic agreement is also difficult to measure as given the diversity of a GP visit a diagnosis is not always available. Other studies have used service utilization (repeat GP visits, subsequent use of other services, doctors’ consultation time) as proxy measures for efficacy or consultation outcome [15, 20]. This may be due to the ease at which these can be measured, but also because they provide a useful quantitative measure of outcome.

The current study sets out to identify the highest level of evidence for telephone consultations in a general practice. The searches only identified one randomized control trial that addressed the question and as such a meta-analysis of numerous studies was not possible, which may have added some weight to the results of the review. However; there were some lower level evidence studies that were identified that were in agreement with the identified randomized control trial.

## Conclusion

Given the minimal research in telephone GP consultations as an alternative for face-to-face GP consultations, it is difficult to make conclusions on the effectiveness of such programmes, especially in a new setting like Australia. From this current evidence, it is likely that GP telephone consults offers an appropriate alternative in some settings. It is important that future research explores the potential for telephone consultations, incorporated with a triage model and the impact this has on service utilization and health outcomes.

## Additional files

Additional file 1: Title of data and Description of file: PRISMA checklist. (DOCX 25 kb)

Additional file 2: Title of data: Search criteria and Supplementary tables. Description of data: This file contains the detail searches, risk of bias results and complete results from McKinstry et al. (DOCX 18 kb)

## Abbreviations

GP: General practitioner
GRADE: Grading of Recommendations Assessment, Development and Evaluation
MeSH: Medical Subject Headings
PRISMA: Preferred Reporting Items for Systematic Reviews and Meta-Analyses
RCT: Randomized control trial
