# Supplementary material for: Telephone consultations for general practice: a systematic review
Source: Syst Rev. 2017 Jul 3;6:128. doi: 10.1186/s13643-017-0529-0 (PMC5496327; doi:10.1186/s13643-017-0529-0)
Supplement: Supplementary file 2 — Title of data: Search criteria and Supplementary tables. Description of data: This file contains the detail searches, risk of bias results and complete results from McKinstry et al. (DOCX 18 kb) [file 13643_2017_529_MOESM2_ESM.docx]

**Search criteria used for a systematic review in telemedicine conducted on the 9^th^ September 2015**

**Ovid MEDLINE(R) 1946 to Present with Daily Update and In-Process & Other Non-Indexed Citations**

(Telemedicine.mp. OR "Tele* Consult*".mp. or "*phone* Consult*".mp. OR exp Telemedicine/ OR exp Remote Consultation/ OR Teleconsult.mp. OR Telehealth.mp. OR ehealth.mp. OR tele-health.mp. OR tele-medicine.mp.) AND (General Practice.mp. OR exp Family Practice/ OR exp General Practice/ OR Family Practi*.mp. OR primary health care.mp. OR exp Primary Health Care/ OR family physician) AND (systematic review*.mp. OR meta analysis.mp OR exp Meta-Analysis/ OR Randomized Controlled Trial.pt. OR exp Randomized Controlled Trials as Topic/ OR exp Randomized Controlled Trial/ OR RCT*.mp)

**CINAHL Plus with Full Text**

(Telemedicine OR Teleconsult OR "Tele* Consult*" or "*phone* Consult*" OR Telephone Consultation* OR Telehealth OR ehealth OR tele-health OR tele-medicine) AND (General Practice OR Family Practi* OR primary health care OR family physician) AND (systematic review OR meta-analysis OR Randomized Controlled Trial OR RCT*)

**The Cochrane Library**

(Telemedicine or "Tele* Consult*" or "*phone* Consult*" or Teleconsult or Telehealth or ehealth or tele-health or tele-medicine or MeSH descriptor: [Telemedicine] explode all trees or MeSH descriptor: [Remote Consultation] explode all trees) AND (General Practice or family physician or Family Practi* or primary health care or MeSH descriptor: [Family Practice] explode all trees or MeSH descriptor: [General Practice] explode all trees or MeSH descriptor: [Primary Health Care] explode all trees) AND (systematic review* or meta analysis or RCT* or Randomized Controlled Trial.pt. or MeSH descriptor: [Randomized Controlled Trials as Topic] explode all trees)

Reviews and trials

**International Clinical Trials Registry Platform**

(Telemedicine OR "Tele* Consult*" OR "*phone* Consult*" OR Teleconsult OR Telehealth OR ehealth OR tele-health OR tele-medicine) AND (General Practice OR Family Practi* OR primary health care OR family physician)

**Table 2: The Cochrane Collaboration’s tool for assessing risk of bias for McKinstry (2002).**

| **Domain** | **Support for judgement** | **Review authors’ judgement** |
| --- | --- | --- |
| *Selection bias.* |  |  |
| **Random sequence generation.** | Describe the method used to generate the allocation sequence in sufficient detail to allow an assessment of whether it should produce comparable groups. | Low |
| **Allocation concealment.** | Describe the method used to conceal the allocation sequence in sufficient detail to determine whether intervention allocations could have been foreseen in advance of, or during, enrolment. | Low |
| *Performance bias.* |  |  |
| **Blinding of participants and personnel***Assessments should be made for each main outcome (or class of outcomes).* | Describe all measures used, if any, to blind study participants and personnel from knowledge of which intervention a participant received. Provide any information relating to whether the intended blinding was effective. | High; as blinding was not possible given that the patients would know which arm they were in as the modes of delivery were so different |
| *Detection bias.* |  |  |
| **Blinding of outcome assessment***Assessments should be made for each main outcome (or class of outcomes)*. | Describe all measures used, if any, to blind outcome assessors from knowledge of which intervention a participant received. Provide any information relating to whether the intended blinding was effective. | Unclear; the results were mainly self-reported (both doctors and patients), so the patients would know which arm they were in, and it was not stated whether analysis would be done blind. |
| *Attrition bias.* |  |  |
| **Incomplete outcome data***Assessments should be made for each main outcome (or class of outcomes).* | Describe the completeness of outcome data for each main outcome, including attrition and exclusions from the analysis. State whether attrition and exclusions were reported, the numbers in each intervention group (compared with total randomized participants), reasons for attrition/exclusions where reported, and any re-inclusions in analyses performed by the review authors. | Low; though for the outcome for patient satisfaction there was significant loss to follow up. |
| *Reporting bias.* |  |  |
| **Selective reporting.** | State how the possibility of selective outcome reporting was examined by the review authors, and what was found. | Low |
| *Other bias.* |  |  |
| **Other sources of bias.** | State any important concerns about bias not addressed in the other domains in the tool.  If particular questions/entries were pre-specified in the review’s protocol, responses should be provided for each question/entry. | Unclear; no conflict of interest was reported and the study was funded by the Scottish Executive Health Department |

Table 3: Outcomes from McKinstry et al (2002)

| **Treatment groups** | **Doctor time/ min (SD)** | **blood test**  **(n/N)** | **Urine test**  **(n/N)** | | **X-rays**  **(n/N)** | **Antibiotics**  **(n/N)** | | **BP measured (n/N)** | **Number of problems**  **(mean (SD), N)** | | **PEI**  **(mean (SD), N)** | **Prepared to use telephone**  **(n/N)** |
| --- | --- | --- | --- | --- | --- | --- | --- | --- | --- | --- | --- | --- |
| **Face-to-face** | 8.2(4.2) | 10/187 | 8/187 | | 5/187 | 30/187 | | 25/188 | 1.2 (0.4) 186 | | 3.0 (3.8) 57 | 39/77 |
| **Telephone** | 6.7(4.9) | 8/181 | 6/181 | | 1/181 | 35/181 | | 12/181 | 1.1 (0.4) 181 | | 2.4 (3.2) 71 | 59/100 |
| **Difference;**  **Mean**  **(95% CI)** | 1.5  (0.6 to 2.4) ^a^ | 0.9%  (-3.5 to 5.3) | 1.0%  (-2.9 to 4.9) | | 2.1%  (-0.4 to 4.7) | -3.3%  (-11.1 to 4.5) | | 6.7%  (0.6 to 12.7)^a^ | 0.0  (0.0 to 0.1) | | 0.6  (-0.6 to 1.8) | -8.4%  (-23.1 to 6.4) |
| **Follow on visits** | | | | | | | | | | | | |
|  | **Subsequent GP contact**  **(mean (SD), N)** | | | **Subsequent OOH contact (mean (SD), N)** | | | **Subsequent A&E contact**  **(mean (SD), N)** | | | **Subsequent tele contact**  **(mean (SD), N)** | | |
| **Face-to-face** | 0.4 (0.7), 188 | | | 0.0 (0.1), 188 | | | 0.0 (0.1), 188 | | | 0.0 (0.1), 188 | | |
| **Telephone** | 0.6 (0.8), 182 | | | 0.0 (0.2), 182 | | | 0.0 (0.2), 182 | | | 0.0 (0.2), 182 | | |
| **Difference;**  **Mean**  **(95% CI)** | -0.2  (-0.3 to -0.0) ^a^ | | | 0.0  (0.0 to 0.0) | | | 0.0  (-0.1 to 0.0) | | | 0.0  (0.0 to 0.0) | | |

^a^ =statistically significant; BP = blood pressure; PEI = patient enablement instrument; GP = general practitioner; OOH = out of hours; A&E = accident and emergency; SD = standard deviation
